# Efficacy and Safety of Liraglutide 3.0 mg in Patients with Overweight and Obese with or without Diabetes: A Systematic Review and Meta-Analysis

**DOI:** 10.1155/2022/1201977

**Published:** 2022-07-19

**Authors:** Mahanjit Konwar, Debdipta Bose, Sanjeet Kumar Jaiswal, Mitesh kumar Maurya, Renju Ravi

**Affiliations:** ^1^Department of Clinical Pharmacology, Seth GS Medical College and KEM Hospital, Mumbai, Maharashtra, India; ^2^Department of Endocrinology, Seth GS Medical College and KEM Hospital, Mumbai, Maharashtra, India; ^3^Department of Clinical Pharmacology, TN Medical College and BYL Nair Charitable Hospital, Mumbai, India; ^4^Department of Clinical Pharmacology, Faculty of Medicine, Jazan University, Jazan, Saudi Arabia

## Abstract

**Background:**

Liraglutide in a 3.0 mg subcutaneous dose daily is approved for weight reduction.

**Objectives:**

Objectives are to evaluate the efficacy and safety of liraglutide 3.0 mg in patients with overweight and obesity irrespective of diabetic status.

**Methods:**

We conducted an electronic database search in PubMed, Embase, and https://ClinicalTrial.gov to identify all randomized control trials (RCTs) that evaluated the efficacy and safety of liraglutide 3.0 mg dose compared to placebo in overweight (≥27 kg/m^2^) and obese (≥30 kg/m^2^) patients above 18 years of age.

**Results:**

We compared the pooled estimate of the study results between liraglutide 3.0 mg groups and placebo groups both in diabetic and nondiabetic patients. The efficacy outcomes that were found to be significant among respective studies involving nondiabetic patients vs. diabetic patients were mean change in body weight from baseline: 12 studies [MD = −5.04 kg (95% CI = −5.60, −4.49), *P* < 0.001, *I*^2^ = 92.95%] vs. 2 studies [MD = −4.14 kg (95% CI = −4.95, −3.32), *P* < 0.001, *I*^2^ = 0%], reduction in waist circumference from baseline: 8 studies [MD = −3.64 cm (95% CI = −4.43, −2.85), *P* < 0.001, *I*^2^ = 96.5%] vs. 2 studies [MD = −3.11 cm (95% CI = −3.88, −2.34), *P* < 0.001, *I*^2^ = 0%], BMI reduction from baseline: 5 studies [MD = −1.95 kg/m^2^ (95% CI = −2.22, −1.68) vs. 1 study [MD = −1.86 kg/m^2^ (95% CI = −2.14, −1.57), *P* < 0.001, *I*^2^ = 0%, *P* < 0.001, *I*^*2*^ = 95.6%], proportion of patients losing more than 5% of weight loss from baseline: 8 studies [RR = 2.21, (95% CI = 1.89, 2.58), *P*=0.03, *I*^2^ = 59.02%] vs. 2 studies [RR = 2.34, (95% CI = 1.93, 2.85), *P*=0.39, *I*^2^ = 0.00%], and 10% weight loss from baseline: 7 studies [RR = 3.36, (95% CI = 1.92, 5.91), *P*=0.00, *I*^2^ = 87.03%] vs. 2 studies [RR = 3.64, (95% CI = 2.46, 5.40), *P*=0.81, *I*^2^ = 0.00%]. Safety outcome assessment with use of liraglutide 3.0 mg compared with placebo in respective nondiabetic vs. diabetic patients revealed significant proportion of patients experiencing the adverse events: 9 studies [RR = 1.11, (95% CI = 1.04, 1.18), *P*=0.00*I*^2^ = 79.15%] vs. 2 studies [RR = 1.06, (95% CI = 1.01, 1.11), *P*=0.42, *I*^2^ = 0.03%] but similar risk of serious adverse events: 9 studies [RR = 1.03, (95% CI = 0.70, 1.51), *P*=0.26, *I*^2^ = 18.54%] vs. 2 studies [RR = 1.11, (95% CI = 0.67, 1.84), *P*=0.25, *I*^2^ = 23.77%] and TDAEs: 4 studies [RR = 0.89, (95% CI = 0.35, 2.28), *P*=0.03, *I*^2^ = 61.89%] vs. 1 study [RR = 2.53, (95% CI = 1.00, 6.37)]. However, the pooled estimates irrespective of the glycaemic status were mean change in body weight from baseline: 14 RCT [MD = −4.91 kg (95% CI = −5.43, −4.39), *P* < 0.001, *I*^2^ = 92.35%], reduction in waist circumference from baseline: 10 studies [MD = −3.55 cm, (95% CI = −4.21, −2.89), *P* < 0.001, *I*^2^ = 94.99%], BMI reduction from baseline: 6 studies [MD = −1.86 kg/m^2^, (95% CI = −2.14, −1.57), *P* < 0.001, *I*^2^ = 96.14%], and proportion of patients losing more than 5% and 10% of weight from baseline: [RR = 2.23, (95% CI = 1.98, 2.52), *P* < 0.001, *I*^2^ = 48.87%] and [RR = 3.28, (95% CI = 2.23, 4.83), *P* < 0.001, *I*^2^ = 78.98%], respectively. Also, the proportion of patients experiencing the adverse event was more with liraglutide 3.0 mg compared with placebo 11 study [RR = 1.09, (95% CI = 1.04, 1.15), *P* < 0.01, *I*^2^ = 76.60%] and similar risk for both serious adverse events: 11 studies [RR = 1.09, (95% CI = 1.04, 1.15), *P* < 0.01, *I*^2^ = 76.60%] and TDAEs: 5 studies [RR = 1.14, (95% CI = 0.50, 2.60), *P* < 0.01, *I*^2^ = 64.93%] with liraglutide compared with placebo.

**Conclusions:**

Liraglutide in 3.0 mg subcutaneous dose demonstrated significant weight reduction with a reasonable safety profile for patients with overweight or obesity regardless of diabetic status compared to placebo.

## 1. Background

Obesity has become a global pandemic that affects diverse communities across lower and upper-middle-income countries [1, 2]. Over the past few decades, the worldwide prevalence of obesity has tripled between 1975 and 2016, with approximately 1.9 billion adults being overweight [3]. Obesity leads to an increased risk of various noncommunicable diseases like diabetes, hypertension, cardiovascular diseases, and cancer and is consequently one of the leading causes of morbidity and mortality worldwide [4–6].

Lifestyle modifications, nutritional counselling, and regular physical activities are effective modalities for weight loss. However, long-term adherence is poor and hence a majority of patients with obesity cannot attain or maintain significant weight loss [7, 8]. Few medications are available for the management of obesity, but safety concerns and questionable long-term efficacy are the major hindrances to their acceptability [9]. Bariatric surgery has shown to have significant weight reduction; the major challenge lies with its approval from the insurance agencies and preoperative procedures [10, 11].

The development of glucagon-like peptide-1 (GLP-1) receptor agonists (GLP-1RA) for the treatment of type 2 diabetes mellitus (T2DM) has opened up a new area for the management of obesity. Liraglutide in subcutaneous doses of 3.0 mg daily is one of the GLP-1 receptor agonists currently available for weight reduction. It was approved by the US-Food and Drug Administration (US-FDA) in December 2014 for chronic weight management as an adjunct to a reduced-calorie diet and increased physical activity in adults with a BMI ≥ 30 kg/m^2^ or a BMI ≥ 27 kg/m^2^ with comorbidities related to weight such as hypertension, diabetes, or dyslipidaemia [12]. The prescribed information also mentions nausea, hypoglycaemia, diarrhoea, constipation, vomiting, headache, decreased appetite, dyspepsia, fatigue, dizziness, abdominal pain, and increased lipase levels as the commonly noted adverse effects with liraglutide. Liraglutide should be used cautiously in patients with acute pancreatitis, acute gall bladder disease, and renal impairment [12].

Several meta-analyses were conducted on the efficacy and safety of the lower doses (lower than 3.0 mg) of liraglutide in diabetes (glycaemic control) and cardiovascular disease (incidence of major adverse cardiovascular events) [13–17]. However, there is paucity of a comprehensive summary of data comparing the efficacy of 3.0 mg of liraglutide concerning relevant parameters related to weight reduction (body weight, BMI, and waist circumference) and the safety of 3.0 mg dose of liraglutide in patients overweight and obese with or without diabetes. Our comprehensive literature search also revealed that none of the preceding meta-analyses which evaluated the impact of liraglutide in weight reduction included both diabetic and nondiabetic participants who are either obese or overweight. Moreover, the preceding meta-analyses evaluated the impact of liraglutide 3.0 mg dose on weight reduction with regard to a few outcome measures such as mean body reduction and 5% and 10% body weight loss. Hence, the present study was envisaged to perform a comprehensive systematic review incorporating a meta-analytic component to evaluate the efficacy and safety of liraglutide as a weight-reducing agent in patients obese and overweight regardless of diabetic status concerning all possible weight reduction-related parameters.

## 2. Materials and Methods

We conducted a study following an a priori study protocol registered with the International Prospective Register of Systematic Reviews-PROSPERO [CRD-42021254137] and the study is reported according to the Preferred Reporting Items for Systematic Reviews and Meta-Analyses (PRISMA) 2020 statement [18].

### 2.1. Data Sources

Two independent reviewers (MK and MM) conducted the literature search on the following databases, PubMed, Embase, and https://ClinicalTrial.gov, using the search terms “ liraglutide,” “Saxenda,” “obesity,” and “randomized controlled trial” from their (PubMed, Embase, and https://ClinicalTrial.gov) inception on December 31, 2021. Additionally, an in-depth manual search was conducted to investigate the relevant references of the retrieved publications. We restricted our search to the English language only. Studies were selected based on the selection criteria given as follows.

### 2.2. Study Selection Criteria

We included randomized controlled trials (RCTs) that evaluated the efficacy of liraglutide 3.0 mg against placebo in patients overweight (body mass index [BMI] ≥27 kg/m^2^ to 30 kg/m^2^) and obese (BMI ≥30 kg/m^2^) above 18 years of age. Nonrandomized studies, studies with active comparators, studies with other doses of liraglutide, and studies with short duration of follow-up and extension studies were excluded. Two independent reviewers (MK and RR) screened all the available studies with relevant keywords. Subsequently, the full texts of the relevant articles were evaluated for eligibility.

### 2.3. Data Extraction

Data were extracted by two independent reviewers (MK and DB) in a self-designed extraction form. Any discrepancies were resolved by consensus in consultation with a third reviewer (RR). The following information was collected from each study and recorded: first author's last name, phase of the trial, study design, sample size, key inclusion criteria, duration of follow-up, mean BMI, nature of behavioural therapy, mean weight loss, mean waist circumference, the proportion of patients with more than 5% and 10% of body weight loss, and proportion of patients with adverse events.

### 2.4. Outcome Measures

The outcome measures are presented for both obese and overweight patients with and without T2DM together as well as separately to explore the overall effect of liraglutide in weight reduction as well as the impact of liraglutide separately in obese and overweight patients with and without T2DM.

### 2.5. Efficacy Outcomes

Efficacy outcome measures were as follows: (a) mean change in body weight, waist circumference, and BMI from baseline and (b) the proportion of patients with at least 5% or 10% loss in body weight from baseline during follow-up.

### 2.6. Safety Outcomes

Safety outcome measures were the proportion of patients with adverse events (AEs), serious adverse events (SAEs), and treatment discontinued due to AEs (TDAEs).

### 2.7. Assessment of Risk of Bias (RoB)

Two investigators (DB and MK) independently evaluated the potential RoB for the methodological quality of included RCTs using the Cochrane Collaboration's tool for assessment of RoB [19]. The RoB was classified into low, high, or unclear risk for the following domains: random sequence generation (selection bias); allocation concealment (selection bias); blinding (performance bias and detection bias); incomplete outcome data (attrition bias); and selective reporting (reporting bias). Any discrepancies that arose had been met through consensus.

### 2.8. Statistical Analysis

Data analysis was performed by two reviewers (MK and DB) using statistical software STATA version 16.0. The pooled risk ratios (RR) and 95% confidence intervals (CI) were calculated by using a random-effects model (restricted maximum likelihood [REML] method) for both efficacy and safety outcomes which were categorical variables [20]. The mean differences with 95% confidence intervals were calculated by using a random-effects model (REML method) for efficacy outcomes which were continuous variables. The efficacy and safety parameters were presented graphically by forest plot. Statistical heterogeneity was estimated through the Higgins *I*^2^ statistics and judged to be either low (<25%), moderate (25%–75%), or high (>75%) [21]. The publication bias was assessed by using Egger's test and presented by using a funnel plot [22]. Tests for funnel plot asymmetry were used only when there are at least 10 studies for assessment. [23]. The level of statistical significance was set at *P* < 0.05.

## 3. Results

### 3.1. Literature Search

**Figure 1:**
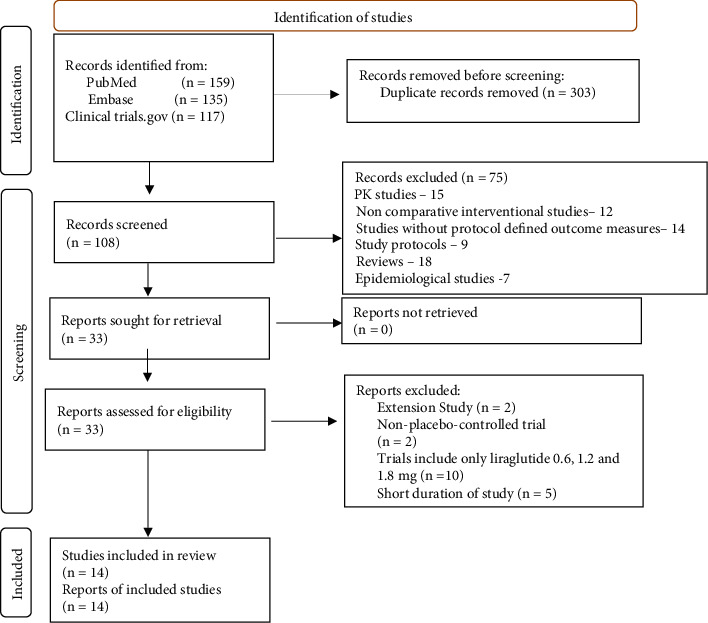
Database searches.

Our primary search from databases yielded a total of 411 studies. On removal of duplicates, we identified 108 articles. Among these, 75 studies were excluded through screening of titles and abstracts based on selection criteria. Subsequently, 33 potentially relevant articles underwent full-text review, and 14 RCTs were included in the meta-analysis. The process of inclusion of articles is summarized in the flow diagram (Figure 1).

### 3.2. Characteristics of the Included Studies

The characteristics of the included studies are summarized in Table 1.

#### 3.2.1. Population

A total of twelve studies were performed exclusively in patients without diabetes [24, 25, 28–37], whereas the remaining studies were conducted in patients with T2DM [26, 27]. Seven studies enrolled participants with a mean BMI ≥27 kg/m^2^ [26–29, 31, 33, 35], and the remaining studies recruited participants with BMI≥ 30 kg/m^2^ [24, 25, 30, 32, 34]. Patients with obesity-related comorbidities were recruited in six out of fourteen studies as outlined in Table 1 [25, 28, 29, 31, 33, 35].

#### 3.2.2. Intervention

The follow-up duration was 52 weeks or more in ten studies [26–28, 30, 32–34] and less than 52 weeks in the remaining studies [24, 25, 29, 31]. Among the fourteen studies, six studies were phase 3 [25–27, 33], four studies were phase 2 [24, 29, 30, 32], three studies were phase 4 [28, 34, 36], and the remaining one was phase 1 [31]. Twelve studies were double-blinded [24–33, 35, 36], and the remaining were open-labelled (Table 1) [34, 37].

#### 3.2.3. Comparator

In six trials, all participants used reduced-calorie intake with physical exercise as an adjunct therapy [24–26, 30, 33, 35]. However, in four trials, the participants received intensive behavioural therapy [27, 34, 36, 37], and the remaining four studies used nutritional/physical training counselling as a supplemental treatment [28, 29, 31, 32] (Table 1).

#### 3.2.4. Outcomes

The primary efficacy outcome was a change in body weight from the baseline in all the studies [24–37]. Ten studies reported a change in waist circumference [24–28, 32, 33, 35–37], and six studies reported a change in BMI [25, 26, 28, 32, 33, 35]. Ten studies reported the proportion of participants achieving at least 5% and/or 10% weight loss from baseline [24, 28, 32, 33, 35–37]. Eleven studies reported the proportion of participants with AEs [24–28, 31–33, 35–37] and SAEs, and five studies reported the data on TDAEs [24, 27, 32, 35, 37].

### 3.3. Risk of Bias (ROB) Assessment

**Figure 2:**
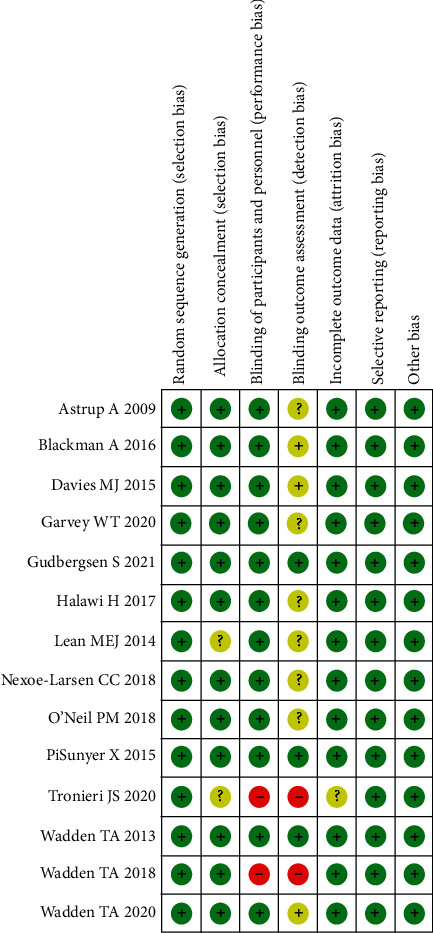
Risk of bias.

Overall, the included studies showed an acceptable methodological quality, with six of them being of excellent quality [25, 26, 28, 33, 35, 37]. Six studies had an unclear risk for blinding of assessors [24, 27, 29–32]. The remaining two studies were of moderate quality as both the participants and study personnel were not blinded [34, 36], and two of the included studies did not mention concealment of allocation [30, 34] (Figure 2).

### 3.4. Synthesis of the Results

#### 3.4.1. Efficacy Outcomes

**Figure 3:**
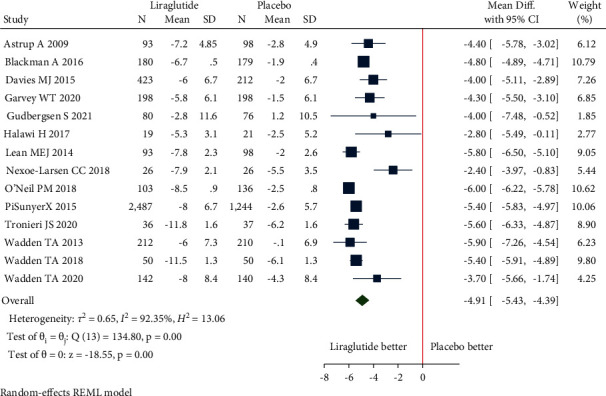
Mean change in body weight (kg).

**Figure 4:**
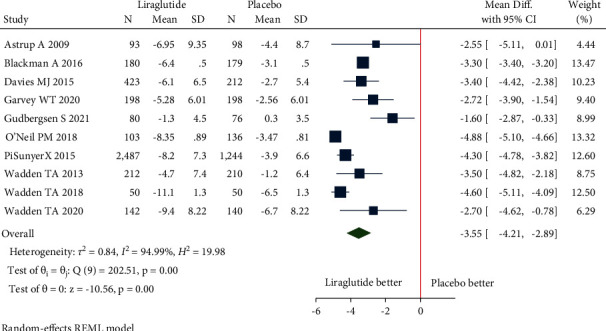
Mean change in waist circumference (cm).

**Figure 5:**
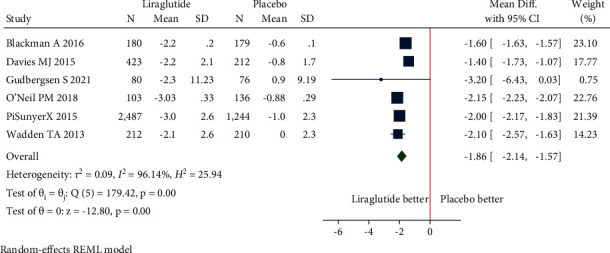
Mean change in BMI (kg/m^2^).

**Figure 6:**
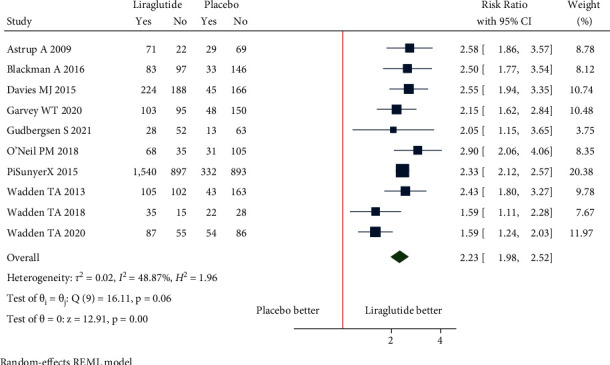
5% weight loss.

**Figure 7:**
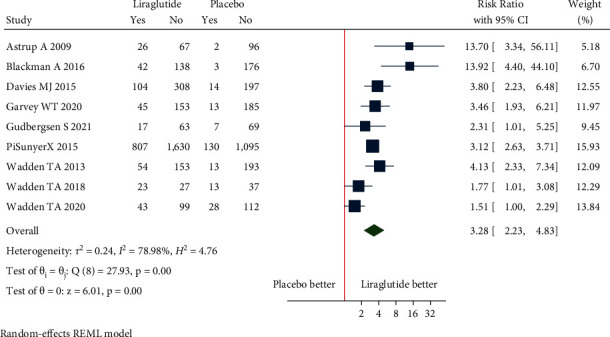
10% weight loss.

(a) Mean change in body weight: the pooled estimate of the fourteen studies showed that liraglutide 3.0 mg resulted in a significant change in body weight from baseline compared to placebo [mean difference (MD) = −4.91 kg (95% CI = −5.43, −4.39), *P* < 0.001, *I*^2^ = 92.35%] (Figure 3).
  - Patients without T2DM: the pooled estimate of the twelve studies showed that liraglutide 3.0 mg resulted in a significant change in body weight from baseline compared to placebo [MD = −5.04 kg (95% CI = −5.60, −4.49), *P* < 0.001, *I*^2^ = 92.95%] (Figure 3).
  - Patients with T2DM: the pooled estimate of the two studies showed that liraglutide 3.0 mg resulted in a significant change in body weight from baseline compared to placebo [MD = −4.14 kg (95% CI = −4.95, −3.32), *P* < 0.001, *I*^2^ = 0%] (Figure 3).
(b) Mean change in waist circumference: the pooled estimate of the ten studies showed that liraglutide 3.0 mg resulted in a significant reduction of waist circumference from baseline compared to placebo [MD = −3.55 cm, (95% CI = −4.21, −2.89), *P* < 0.001, *I*^2^ = 94.99%] (Figure 4).
  - Patients without T2DM: the pooled estimate of the eight studies showed that liraglutide 3.0 mg resulted in significant reduction in waist circumference from baseline compared to placebo [MD = −3.64 cm (95% CI = −4.43, −2.85), *P* < 0.001, *I*^2^ = 96.5%] (Figure 4).
  - Patients with T2DM: the pooled estimate of two studies showed that liraglutide 3.0 mg resulted in significant reduction in waist circumference from baseline compared to placebo [MD = −3.11 cm (95% CI = −3.88, −2.34), *P* < 0.001, *I*^2^ = 0%] (Figure 4).
(c) Mean change in BMI: the pooled estimate of the six studies showed that liraglutide 3.0 mg use was associated with a significant reduction of BMI from baseline compared to placebo [MD = −1.86 kg/m^2^, (95% CI = −2.14, −1.57), *P* < 0.001, *I*^2^ = 96.14%] (Figure 5).
  - Patients without T2DM: the pooled estimate of the five studies showed that liraglutide 3.0 mg resulted in a significant reduction in BMI from baseline compared to placebo [MD = −1.95 kg/m^2^ (95% CI = −2.22, −1.68), *P* < 0.001, *I*^2^ = 95.6%] (Figure 5).
  - Patients with T2DM: the one study showed that liraglutide 3.0 mg resulted in a significant reduction in BMI from baseline compared to placebo [MD = −1.40 kg/m^2^ (95% CI = −1.73, −1.07), *P* < 0.001, *I*^2^ = NA] (Figure 5).
(d) 5% weight loss: the pooled estimate of eight studies in nondiabetic patients [RR = 2.21, (95% CI = 1.89, 2.58), *P*=0.03, *I*^2^ = 59.02%] and two studies in diabetic patients [RR = 2.34, (95% CI = 1.93, 2.85), *P*=0.39, *I*^2^ = 0.00%] revealed significant proportion of patients losing more than 5% of weight loss from baseline with administration of liraglutide 3.0 mg when compared with placebo (Figure 6). The pooled estimate of the ten studies demonstrated that liraglutide 3.0 mg resulted in significantly higher proportion of participants achieving at least 5% weight loss from baseline compared to placebo [RR = 2.23, (95% CI = 1.98, 2.52), *P* < 0.001, *I*^2^ = 48.87%] (Figure 6).
(e) 10% weight loss: the pooled estimate of seven studies in nondiabetic patients [RR = 3.36, (95% CI = 1.92, 5.91), *P*=0.00, *I*^2^ = 87.03%] and two studies in diabetic patients [RR = 3.64, (95% CI = 2.46, 5.40), *P*=0.81, *I*^2^ = 0.00%] revealed significant proportion of patients losing more than 10% of weight from baseline with administration of Liraglutide 3.0 mg when compared with placebo (Figure 7). The pooled estimate of the nine studies demonstrated that liraglutide 3.0 mg resulted in significantly higher proportion of participants achieving at least 10% weight loss from baseline compared to placebo [RR = 3.28, (95% CI = 2.23, 4.83), *P* < 0.01, *I*^2^ = 78.98%] (Figure 7).

#### 3.4.2. Safety Outcomes

**Figure 8:**
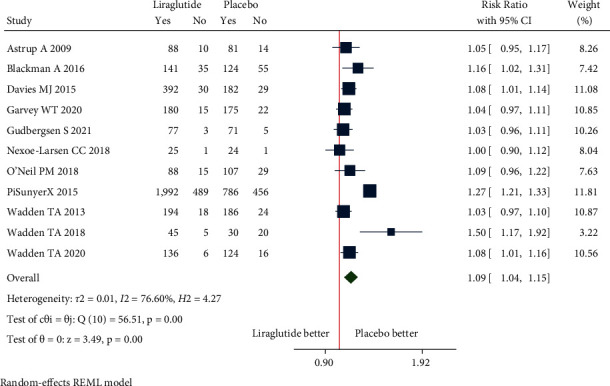
Adverse events.

**Figure 9:**
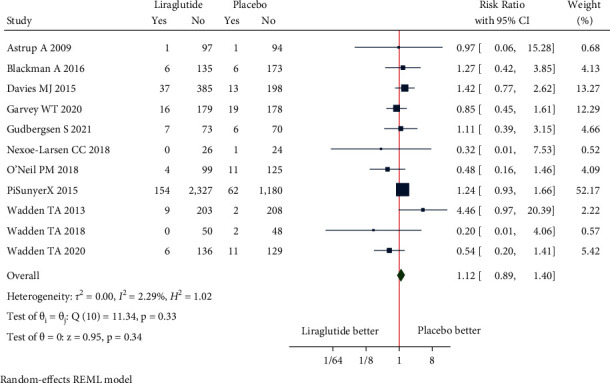
Serious adverse events.

**Figure 10:**
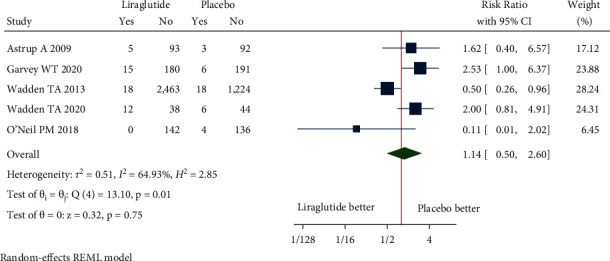
Treatment discontinuation due to adverse events.

- Adverse events: the pooled estimate of nine studies in nondiabetic patients [RR = 1.11, (95% CI = 1.04, 1.18), *P*=0.00, *I*^2^ = 79.15%] and two studies in diabetic patients [RR = 1.06, (95% CI = 1.01, 1.11), *P*=0.42, *I*^2^ = 0.03%] revealed significant proportion of patients experiencing the adverse events in liraglutide 3.0 mg group when compared with placebo (Figure 8). The pooled estimate of the eleven studies showed that liraglutide 3.0 mg had higher risk of AEs compared to placebo [RR = 1.09, (95% CI = 1.04, 1.15), *P* < 0.01, *I*^2^ = 76.60%] (Figure 8).
- Serious adverse events: the pooled estimate of nine studies in nondiabetic patients [RR = 1.03, (95% CI = 0.70, 1.51), *P*=0.26, *I*^2^ = 18.54%] and 2 studies in diabetic patients [RR = 1.11, (95% CI = 0.67, 1.84), *P*=0.25, *I*^2^ = 23.77%] revealed similar risk of SAE experienced with use of liraglutide 3.0 mg when compared with placebo (Figure 9). The pooled estimate of the eleven studies showed that liraglutide 3.0 mg had similar risk of SAEs compared to placebo [RR = 1.12, (95% CI = 0.89, 1.40), *P*=0.33, *I*^2^ = 2.29%] (Figure 9).
- Treatment discontinuation due to AEs: the pooled estimate of four studies in nondiabetic patients [RR = 0.89, (95% CI = 0.35, 2.28), *P*=0.03, *I*^2^ = 61.89%] and one study in diabetic patients [RR = 2.53, (95% CI = 1.00, 6.37)] revealed treatment discontinuations due to adverse events [TDAEs] to be similar between liraglutide 3.0 mg group and the placebo-controlled arm (Figure 10). The pooled estimate of the five studies showed that liraglutide 3.0 mg had similar risk of TDAEs compared to placebo [RR = 1.14, (95% CI = 0.50, 2.60), *P*=0.01, *I*^2^ = 64.93%] (Figure 10).

### 3.5. Publication Bias

**Figure 11:**
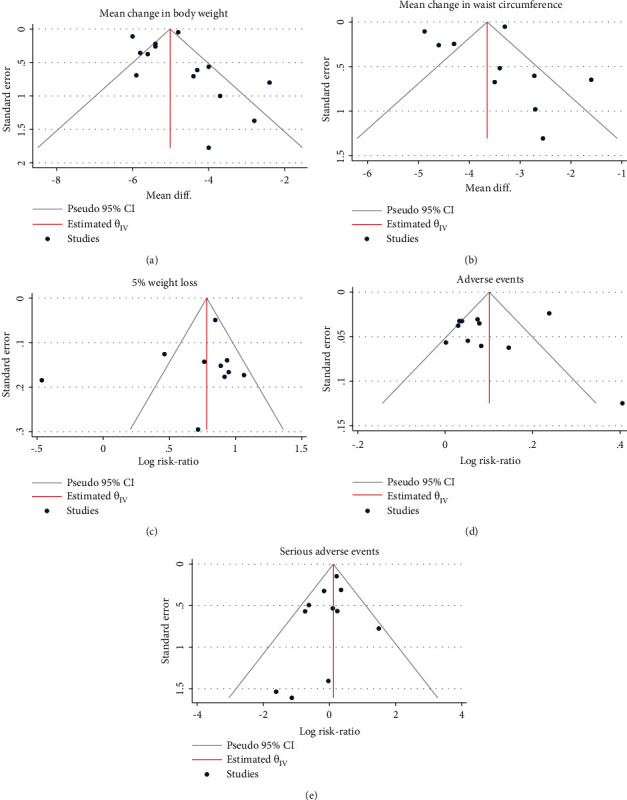
Funnel plot. (a) Mean change in body weight. (b) Mean change in waist circumference. (c) 5% weight loss. (d) Adverse events. (e) Serious adverse events.

Visual analysis of funnel plots showed the presence of publication bias for mean change in body weight and waist circumference and RR for 5% weight loss which was confirmed in quantitative analysis with Egger's test (*P*=0.005, *P*=0.03, and *P*=0.0013, respectively) (Figures 11(a)–11(e)).

## 4. Discussion

In our systematic review and meta-analysis, data from fourteen RCTs were evaluated to summarize the evidence regarding the efficacy and safety of liraglutide 3.0 mg for the treatment of patients with BMI greater than or equal to 27 kg/m^2^. The study has several key findings. First, liraglutide in subcutaneous doses of 3.0 mg had shown a significant reduction in body weight (mean reduction of 4.9 kg), waist circumference (mean reduction of 3.5 cm), and BMI (mean reduction of 1.86 kg/m^2^) from baseline compared to placebo. Second, a significantly higher proportion of participants has achieved at least 5% and 10% weight loss from baseline compared to placebo. Third, liraglutide 3 mg was associated with a higher risk of AEs but was associated with similar risk of SAEs and TDAEs compared to placebo.

Liraglutide, with 97% structural homology to human GLP-1, delays gastric emptying and induces satiety, leading to decreased energy intake and weight reduction [38]. The underlying mechanisms that mediate the effects of weight loss of liraglutide are most probably a combination of effects on the gastrointestinal tract and brain [38]. A study conducted by van Can et al. demonstrated that liraglutide-induced weight loss appeared to be mediated by reduced appetite and energy intake rather than increased energy expenditure [39].

A systematic review and meta-analysis by Khera et al. demonstrated that liraglutide, with at least one year of treatment, was associated with significant weight loss compared to placebo [40]. Additionally, treatment with liraglutide was associated with higher odds of adverse event-related treatment discontinuation when compared with placebo [40]. Data from the trials of the SCALE program, Le Roux et al. have shown significantly greater weight loss with liraglutide 3.0 mg, compared to placebo, in patients with BMI above and below 35 kg/m^2^ [41]. However, there was no evidence that the weight-lowering effect of liraglutide 3.0 mg differed and the safety profile was broadly similar across BMI subgroups [41]. Another meta-analysis by Singh and Singh illustrated a significant reduction in body weight with liraglutide 3.0 mg and opined that it should be the preferred agent for weight reduction in obese patients with T2DM [42]. Another meta-analysis by Zhang et al. observed higher odds of weight loss with liraglutide 3.0 mg in obese patients without diabetes with a higher proportion of patients who discontinued treatment due to adverse events, compared to placebo [43]. The current study noted similar efficacy and safety of 3.0 mg dose of liraglutide in weight reduction in both diabetic and nondiabetic individuals with obesity and overweight, as shown in the other three meta-analyses. However, the number of studies that are included in the present analysis is higher than the previous meta-analyses. Moreover, the present analysis comprehensively analysed the efficacy of 3.0 mg dose of liraglutide in all the possible parameters (mean weight reduction, BMI reduction, waist circumference reduction, and 5% and 10% weight loss) related to weight reduction, unlike the previous meta-analyses where the efficacy outcome measures were restricted to mostly mean weight reduction along with 5% weight loss. The present meta-analyses also included both the population with or without diabetes, unlike the previous meta-analyses where the included studies were either on diabetic patients or participants without diabetes. The safety analysis of the present study noted similar observations as found in the other meta-analyses but in the Zhang et al. study the incidence of TDAEs was similar between the liraglutide 3.0 mg and placebo group which could be due to the inclusion of studies with only nondiabetic participants, unlike the present analysis where studies with both diabetic and nondiabetic participants were included.

The US Preventive Services Task Force (2018) and Canadian Adult Obesity Clinical Practice Guidelines (2020) recommend referral of all obese patients to comprehensive, intensive, multicomponent interventions including psychological interventions, pharmacological therapies, and bariatric surgical procedures [44, 45]. The European Association for the Study of Obesity (EASO) also endorses the use of approved weight-loss medications for long-term weight maintenance to ameliorate comorbidities and to enhance adherence to behavioural changes [46]. However, there are no current recommendations to guide clinicians regarding the choice of individual drugs for the management of obesity. The network meta-analysis from Khera et al. demonstrated that the phentermine-topiramate was associated with the highest probability of achieving at least 5% weight loss followed by liraglutide. Similarly, phentermine-topiramate was associated with the highest probability of achieving at least 10% weight loss followed by liraglutide [40]. However, considering the fact that phentermine-topiramate label carries a risk evaluation and mitigation strategy, liraglutide could be a safer option as many of the potential recipients will have multiple comorbidities. Hence, we suggest that liraglutide in subcutaneous doses of 3.0 mg could be an appropriate first-line agent in obese or overweight people regardless of diabetes status who need pharmacotherapy as an adjunct to lifestyle modification, especially for the diabetic patients, patients with cardiovascular risk factors, uncontrolled hypertension, or a history of heart disease, where sympathomimetics are contraindicated. Additionally, treatment with liraglutide was associated with higher odds of adverse event-related treatment discontinuation when compared with placebo. However, given the differences in efficacy, safety, and interindividual variation in drug response, the ideal approach to weight loss should be highly individualized, identifying appropriate drug, behavioural interventions, and surgical procedures [47]. Nevertheless, short-term studies may not provide comprehensive information on the long-term safety and effectiveness of liraglutide, and hence rigorous post-marketing surveillance studies are warranted.

To the best of our knowledge, this is the first meta-analysis which looked at the overall efficacy and safety of liraglutide 3.0 mg dose in obese and overweight patients with or without T2DM and also explored the impact of liraglutide separately in patients with or without T2DM. However, our meta-analysis has certain limitations that should be taken into account. First, some of the included RCTs were of small sample size with a short duration of follow-up; consequently, the impact on weight reduction-related parameters might be overestimated as the similar efficacy may not be sustainable if the duration of follow-up longer. Second, there were considerable differences among the studies in patient characteristics, co-interventions/background therapy, study design, and duration of follow-up, leading to significant heterogeneity. Furthermore, we have included published articles from only two bibliographic databases, and we could not include the data from grey literatures (unpublished studies and dissertations and conference proceedings) which are not publicly available. Hence, the results may not fully reflect the existing evidential base.

In summary, liraglutide in 3.0 mg subcutaneous dose demonstrated a significant weight reduction with a reasonable safety profile for patients with overweight or obesity with or without T2DM. Thus, liraglutide can be a good candidate to be included as a first-line pharmacotherapeutic agent for the management of obesity as an adjunct to lifestyle modification regardless of diabetic status.

## Figures and Tables

**Table 1:** Characteristics of studies.

Study	Phase	Trial design	Key inclusion criteria	T2DM	Duration (weeks)	Sample size lira/placebo		Mean BMI (SD) lira/placebo at baseline		Diet/behavioural therapy
Astrup et al. [24]	2	Double-blind	18–65 years, with BMI of 30–40 kg/m², and FPG of less than 7 mmol/L at the run-in period	No	20	93	98	34·8 (2·8)	34.9 (2.8)	Reduced-calorie diet and maintain or increase physical activity
Blackman et al. [25]	3	Double-blind	18–64 years with BMI ≥30 kg m^2^ diagnosed with moderate/severe OSA	No	32	180	179	38.9 (6.4)	39.4 (7.4)	Reduced-calorie diet and increased physical activity
Davies et al. [26]	3	Double-blind	Age 18 years or older with BMI of ≥27.0 taking 0 to 3 oral hypoglycaemic agents and HbA1c level 7.0% to 10.0%	Yes	56	423	212	37.1 (6.5)	37.4 (7.1)	Reduced-calorie diet and increased physical activity
Garvey et al. [27]	3	Double-blind	Aged ≥18 years and BMI ≥27 kgm^2^ with an ≥HbA1c 6.0 to ≥10% and T2DM treated with basal insulin and less than or equal to two OADs	Yes	56	198	198	35.9 (6.5)	35.3 (5.8)	Intensive behaviour therapy
Gudbergsen et al. [28]	4	Double-blind	Aged 18 and 74 y with BMI ≥27 kg m^2^ with knee osteoarthritis	No	52	80	76	32.8 (5.5)	31.3 (4.0)	Nutritional counselling
Halawi et al. [29]	2	Double-blind	Aged 18 and 65 years with BMI ≥27.0 kg/m^2^, with an obesity-related comorbidity, and adults with obesity (BMI >30 kg/m²),	No	26	19	21	37.2 (7.5)	34.6 (2.6)	Dietary and exercise interventions
Lean et al. [30]	2	Double-blind	Aged 18–65 yrs with BMI ≥30 and ≤40 kgm and FPG <7.0 mmolL	No	52	93	98	34.8 (2.8)	34.9 (2.8)	Reduced-calorie diet and increased physical activity
Nexøe-Larsen et al. [31]	1	Double-blind	Aged 18 and 64 years with BMI ≥27.0 kg/m^2^, ultrasound assessment of gallbladder volume of acceptable quality at screening overweight and obese subjects	No	12	26	26	32.5 (3.6)	32.6 (3.3)	Nutritional and physical activity counselling
O'Neil et al. [32]	2	Double-blind	Aged ≥18 years and BMI ≥30 kgm^−2^	No	52	103	136	38·6 (6·6)	40·1 (7·2)	Dietary and exercise interventions
Pi-Sunyer et al. [33]	3	Double-blind	Aged ≥18 years and BMI ≥30 kgm^−2^ or ≥27 kgm^2^ with comorbidities of treated or untreated dyslipidaemia and/or treated or untreated hypertension	No	56	2487	1244	38.3 (6.4)	38.3 (6.3)	Reduced-calorie diet and increased physical activity
Tronieri et al. [34]	4	Open label	Aged 21–70 years with BMI ≥30 and ≤55 kg/m² and had no serious medical or psychological conditions	No	52	36	37	39.2 (5.0)	37.6 (4.1)	Intensive behaviour therapy
Wadden et al. [35]	3	Double-blind	Aged ≥18 years with BMI ≥30 kgm^2^ or ≥27 kgm^2^ with comorbidities of treated or untreated dyslipidaemia and/or treated or untreated hypertension	No	56	212	210	38.3 (6.4)	38.3 (6.3)	Reduced-calorie diet and increased physical activity
Wadden et al. [36]	4	Open label	Aged 21–70 years with BMI of 30–55 kg/m^2^; prior lifetime weight-loss effort with diet and exercise	No	52	50	50	38.5 (5.4)	38.0 (4.3)	Intensive behaviour therapy
Wadden et al. [37]	3	Double-blind	Aged ≥18 years, with BMI ≥30 kg/m^2^	No	56	142	140	39.3 (6.8)	38.7 (7.2)	Intensive behaviour therapy

## Data Availability

No data were used to support this study.
